# Detecting small plant peptides using SPADA (Small Peptide Alignment Discovery Application)

**DOI:** 10.1186/1471-2105-14-335

**Published:** 2013-11-20

**Authors:** Peng Zhou, Kevin AT Silverstein, Liangliang Gao, Jonathan D Walton, Sumitha Nallu, Joseph Guhlin, Nevin D Young

**Affiliations:** 1 Department of Plant Pathology, University of Minnesota, St. Paul, Minnesota 55108, USA; 2 Supercomputing Institute for Advanced Computational Research, University of Minnesota, Minneapolis, Minnesota 55455, USA; 3 Department of Plant Biology and U.S. Department of Energy Plant Research Laboratory, Michigan State University, East Lansing, Michigan 48824, USA; 4 Department of Ecology and Evolution, University of Chicago, Chicago, Illinois 60637, USA; 5 Department of Plant Biology, University of Minnesota, St. Paul, Minnesota 55108, USA

**Keywords:** Protein family, Genome annotation, Homology search, Gene prediction

## Abstract

**Background:**

Small peptides encoded as one- or two-exon genes in plants have recently been shown to affect multiple aspects of plant development, reproduction and defense responses. However, popular similarity search tools and gene prediction techniques generally fail to identify most members belonging to this class of genes. This is largely due to the high sequence divergence among family members and the limited availability of experimentally verified small peptides to use as training sets for homology search and *ab initio* prediction. Consequently, there is an urgent need for both experimental and computational studies in order to further advance the accurate prediction of small peptides.

**Results:**

We present here a homology-based gene prediction program to accurately predict small peptides at the genome level. Given a high-quality profile alignment, SPADA identifies and annotates nearly all family members in tested genomes with better performance than all general-purpose gene prediction programs surveyed. We find numerous mis-annotations in the current *Arabidopsis thaliana* and *Medicago truncatula* genome databases using SPADA, most of which have RNA-Seq expression support. We also show that SPADA works well on other classes of small secreted peptides in plants (e.g., self-incompatibility protein homologues) as well as non-secreted peptides outside the plant kingdom (e.g., the alpha-amanitin toxin gene family in the mushroom, *Amanita bisporigera*).

**Conclusions:**

SPADA is a free software tool that accurately identifies and predicts the gene structure for short peptides with one or two exons. SPADA is able to incorporate information from profile alignments into the model prediction process and makes use of it to score different candidate models. SPADA achieves high sensitivity and specificity in predicting small plant peptides such as the cysteine-rich peptide families. A systematic application of SPADA to other classes of small peptides by research communities will greatly improve the genome annotation of different protein families in public genome databases.

## Background

A major challenge in translating new genome sequences into useful community resources is the accurate annotation of genes and other functionally-relevant features [1]. While there have been clear improvements in gene prediction algorithms [2], accurate prediction of small one and two-exon genes remains stubbornly problematic [3]. False-positive signals arising from the poor specificity of promoter motifs and other commonly-used signals employed by general purpose gene-finding algorithms are widespread [4-7]. To address the flood of false-positive signals for small genes, many annotators filter out small-gene predictions lacking direct experimental expression evidence, resulting in a major problem of false negatives [3,4].

We propose here an alternative and complementary strategy for genome-wide annotation – a strategy that has as its strength predicting the small one- and two-exon genes that all-purpose gene-finding algorithms often fail to predict accurately. Our approach focuses on finding all related paralogous genes within a target gene family and then using signals from the corresponding multiple sequence alignment to aid in refining the model predictions. We have implemented this approach in an open-source and freely available application called SPADA (Small Peptide Alignment Discovery Application). SPADA can be used directly with a user’s own protein family alignments or with a comprehensive set of protein family alignments from public sources such as Pfam [8], InterPro [9] or PROSITE [10], enabling the exhaustive discovery of essentially all members of the input families within a given genome sequence. Because these public resources continue to expand and include new and novel protein families, SPADA’s ability to comprehensively identify arbitrarily large families of small peptides in genomes will steadily grow.

Here we describe the conceptual basis of SPADA and go onto test its performance with selected families of notoriously difficult genes to annotate properly - specifically, plant Cysteine-Rich Peptides (CRPs) in two model plant species (*Arabidopsis thaliana* and *Medicago truncatula*), the S-Protein homologue (SPH) family in *A. thaliana*, and the alpha-amanitin toxin gene family in the mushroom *Amanita bisporigera*. In the case of CRPs, we examine the accuracy and recall compared to published composite test/training sets for these species based on previous semi-manual curation and subsequent experimental expression validation [11-13]. We also compare SPADA’s performance against a range of commonly-used generic gene-prediction algorithms [14-17], providing evidence of SPADA’s advantage in identifying these challenging classes of small peptides.

## Method

**Figure 1:**
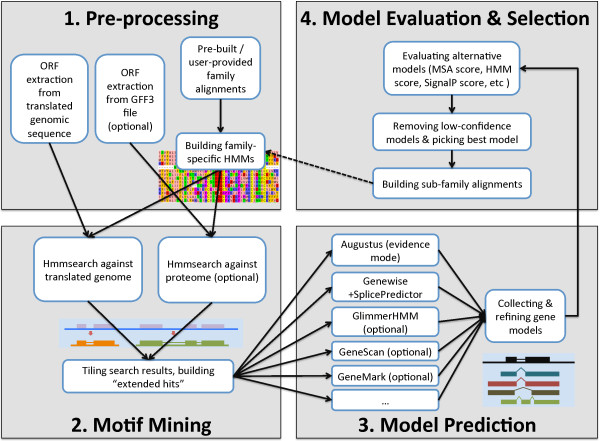
The SPADA workflow.

SPADA is a computational pipeline that, when provided with a multiple sequence alignment for a gene/protein family of interest, identifies all members of this family in a target genome. Technically, SPADA’s pipeline is a general homology-based gene finding program with specifically enhanced power to detect and annotate small peptides with one or two exons. Unlike general-purpose gene prediction programs such as Fgenesh [18], SPADA works on an entire gene family at one time - with the goal of finding all family members in the genome. Unlike other homology-based gene predictors such as Genewise [19] and Exonerate [20] that map a single protein sequence to the target genome, SPADA performs a similarity search using a profile alignment and identifies all homologs of the family. In addition, SPADA provides automated access to both similarity search tools (e.g., BLAST [21] and HMMER [22]) and *ab initio* gene predictors (e.g., Augustus), significantly improving the annotation efficiency of multi-member gene families. As shown in Figure 1, SPADA consists of four consecutive components: 

• Pre-Processing

• Motif Mining

• Model Prediction

• Model Evaluation & Selection

In SPADA, HMMER [22] is first used to identify hits in the target genome sequence (translated in six reading frames) as well as in the target proteome (if available) using a reasonably generous E-value (10). These hits are then tiled with regard to their genomic coordinates and merged into overlapping clusters. Finally, one best hit in each cluster is picked to generate a list of candidate hits.

The pipeline then allows the user to run one or more processes to predict gene structures for these potential genes. By default, SPADA runs Augustus [17] using hit locations as clues for "CDS regions" (coding sequence). In parallel, SPADA runs a custom pipeline optimized for predicting the exon boundaries of genes containing one or two exons using GeneWise [19], SplicePredictor [23] and custom Perl scripts.

In the next step, all gene structure predictions are combined to make a raw calling set, with each hit having one or more gene structure predictions. SPADA uses multiple statistics to assess the confidence of each candidate gene model, including an alignment score (mean pairwise score with known members in the original family-specific multiple sequence alignment), an HMM alignment score (sum of posterior probability scores in the Hmmsearch output file), the presence/absence of proper start/stop codons, as well as the SignalP D-score [24] in the case of secreted peptides.

Finally, the best candidate gene model is picked for each hit and the resulting set is filtered using empirical cutoffs (hmmsearch E-value of 0.001) to remove false positives.

### Pre-processing

#### Building family-specific multiple sequence alignments

The original motivation for developing this pipeline was accurately identifying and predicting Cysteine-Rich Peptides (CRPs) in plant genomes. For this purpose, SPADA comes with a complete set of manually-curated protein sequence alignments for plant Cysteine-Rich Peptide (CRP) families [11]. In 2007, Silverstein *et al.* built multiple sequence alignments for most plant CRP families through iteratively scanning EST sequences from different plant species in TIGR’s Gene Indices [11]. These alignments were re-aligned here using ClustalO [25] and trimmed using trimAl [26] to remove spurious sequences and poorly aligned positions. Finally, a profile Hidden Markov Model (HMM) was built for each CRP family using "hmmbuild" in the HMMER package [27].

As a general homology-based gene finding program, SPADA has been designed to work with any set of protein families. Users can start with a list of amino acid sequence alignments of their own interest, run the script "build_profile.pl" to generate custom HMM profiles, and initiate the pipeline using the new HMM(s). With this in mind, we have tested SPADA’s performance on an additional protein family as a proof of concept (see Results section) and assessed its applicability to secreted protein families other than CRPs.

#### Processing genome sequence and annotation

In SPADA, genome FASTA sequences are translated in all six reading frames to amino acid sequences and then Open Reading Frames (ORFs) are extracted by breaking up these long amino acid sequences using stop codons. Here an ORF is defined as a segment of amino acid sequence with at least 15 residues and uninterrupted by stop codons. Extracting ORFs from the original translated genomic sequence reduces the target database size for the subsequent motif mining step and improves sensitivity. Using ORFs also ensures that no protein-coding exon spans stop codons in the middle of a sequence and that each exon will have a reasonable length. In theory, all protein-coding exons should locate within these ORFs, which will be discovered in the next motif-mining step. However, the exact exon boundaries are still unclear at this point of the search procedure.

If a gene annotation file in General Feature Format version 3.0 (GFF3) is available, SPADA can also read and process it, extracting the amino acid sequences of existing annotations and passing them onto the next motif mining step. In doing so, exon boundaries can be better refined, further improving the accuracy in the model prediction step.

Hard-masking of genome sequences is recommended (replacing repetitive sequences with 'N’s) before running the pipeline. Some plant species have very large genomes with highly repetitive content (e.g., Maize [28]). By hard-masking the genome sequence, the target database size in the motif-mining step is effectively reduced, significantly improving the search sensitivity of the entire pipeline. However, if many family members locate in repeat-rich genomic regions (such as the fungi effector families [29]), the unmasked genome version should be used.

### Motif mining

In SPADA, profile HMMs are used to search against translated genomic sequences (and known protein sequences, if available) using "hmmsearch", a component of the HMMER package (v3.0) [27]. This program finds significant hits against a protein sequence database using one or more profiles as inputs. The output of the scan is a list of genomic intervals with significant sequence similarity to query profiles and amino acid sequences translated from these intervals. For single-exon genes, a contiguous stretch of amino acid sequence in the target databases will be discovered, roughly corresponding to the exon in the original genomic sequence. For genes containing two or more exons, partial amino acid sequence hits corresponding to different exons will be separated by introns (if they share a reading frame) or distributed in different target sequences (if in different reading frames). SPADA collects all these full and partial hits in translated protein sequences, recovers their original genomic coordinates, filters out low-significance hits (E-value lower than 0.1), selects the most significant hit for each genomic interval since multiple input profiles may hit the same region, and merges nearby partial hits. During this merging step, SPADA requires that each neighboring partial hit should hit a different segment (either upstream or downstream) in the input profile HMM. The merged genomic intervals (called "extended hits") roughly correspond to the multiple exons in the underlying gene model - although the exact intron-exon boundaries and start/stop codon locations are yet to be refined at this stage of the procedure.

In parallel, SPADA searches against existing protein sequences (generated using the GFF3 annotation file), yielding a separate list of hits to the input profiles. These hits are also treated as partial hits, i.e., mapped to their original genomic coordinates and then used to build "extended hits". This "hmmsearch against proteome" step is considered complementary to the abovementioned "hmmsearch against translated genome" step, since it improves prediction sensitivity by capturing otherwise non-significant partial hits in the translated genome search.

### Model prediction

At this point, SPADA has generated a list of "extended hits" approximately corresponding to actual exon boundaries. For each extended hit the surrounding genomic sequence is extracted. By default, 2500 bp upstream from the hits are extracted, since the first exon (containing the signal peptide) is usually separated from the second exon (with the mature peptide) by an intron up to 1500 bp, as determined by manual curation and understanding of plant genomes. At the other end, 1500 bp downstream from the hit boundaries are extracted, since the correct stop codon can typically be found within 1000 bp downstream of the HMM hit [11]. SPADA next runs one or more components (selected by the user) in parallel to determine gene structure in this region. A total of five prediction components are currently supported by the pipeline: Augustus [17], GeneWise [19], GlimmerHMM [14], GeneMark [15] and GeneID [16]. By default SPADA only runs two of these components (Augustus and GeneWise) since performance evaluation on a group of common plant peptides suggests that running all five of them does not offer a significant extra gain compare to running just two of them (see Results & Discussion).

The first component, which we denote "Augustus_evidence", runs Augustus [17] in its "evidence mode". The genomic sequence is used as input along with a "hint file" providing the program instructions for which part(s) of the input sequence are known to be part(s) of the coding sequence. In other words, location information of extended hits is incorporated in the prediction process. Augustus will then try to complete the gene model by looking for start/stop codons and canonical donor-acceptor splice sites around the hits while preserving the open reading frame. The improvement in prediction accuracy and specificity by running Augustus in the "evidence mode" (as compared to the "Augustus *de novo* mode") is significant and will be discussed in Results & Discussion.

In parallel, SPADA runs a custom pipeline specifically designed to identify and predict genes with one or two exon(s) and with a leading signal peptide. This component, which we denote "Genewise+SplicePredictor", first runs GeneWise [19] to align the extended hit sequence (translated to amino acid sequence) to genomic sequence and identifies compatible splice sites that preserve the hit ORFs. If GeneWise fails due to non-canonical splice sites, SPADA then runs SplicePredictor [23] to find all possible donor/acceptor splice sites and extracts compatible ones, extending the ORFs to the nearest start codon and stop codon. In practice, this "Genewise+SplicePredictor" approach works well as a complement to the "Augustus_evidence" approach (see Results and Discussion).

At this stage in the pipeline, SPADA reports all compatible gene models predicted by the two components. These candidate models are then passed on to the next step for evaluation in order to generate a best calling set.

### Model evaluation & selection

For each extended hit, SPADA then evaluates the underlying candidate models using a number of measures and picks the most "confident" model for output. These evaluation statistics include the presence of start/stop codons at the beginning/end of the model, the presence of inframe stop codons, the SignalP score [24] in the case of a secreted gene family, and in particular, the Multiple Sequence Alignment (MSA) score and the "Hmmsearch Probability" (HmmProb) score, as described below.

In theory, the correct gene model should encode an amino acid sequence that aligns to the original family-specific protein alignments better than any other candidate models. To calculate the MSA score, SPADA aligns the amino acid sequence of the candidate model to the profile alignment using ClustalO "profile-to-profile" mode [25]. SPADA then scores all pairwise alignments using BLOSUM80 scoring matrix [30] and calculates a mean alignment score. The BLOSUM80 matrix is used instead of BLOSUM62 because the sequences that are being aligned tend to be fairly similar to each other, and a matrix with more conserved target frequencies such as BLOSUM80 should be more reasonable. Ideally, the candidate model with the highest MSA score should be the most probable model.

Nevertheless, the MSA score is not sufficient to pick the best model, since candidate models are sometimes too close to each other in sequence and the MSA scores may not vary appreciably among model alternatives. Therefore SPADA also calculates an "Hmmsearch Probability" Score for each candidate model. In theory, if hmmsearch is run using the original family HMM against all candidate models, the most significant hit in the output should then be the best model. In practice, the probability score in the hmmsearch output serves as a better predictor than the E-value itself, especially when a model contains more than one hit domain. The MSA score and the HmmProb score are used to evaluate each candidate model. SPADA then picks the best candidate model that meets the following criteria: (1) it has a SignalP D-score of no less than 0.4 (determined according to the software manual, this option could be turned off to allow prediction of non-secreted gene families); (2) it has proper start/stop codons and no premature stop codon; and (3) it has the highest (MSA score + HmmProb score).

SPADA uses a relatively relaxed E-value cutoff in the motif mining step (e.g., 10 for running hmmsearch) in order to increase specificity. This also results in numerous false positive hits. These hits will generally not have valid candidate gene models built for them in the model prediction step, and thus would not make it into the ultimate output. However, SPADA does employ a final filtering step based on hmmsearch E-value to refine gene models that are retained. We evaluated the performance of the pipeline under different final E-value cutoffs (see Results and Discussion) and set the default cutoff to 0.001, which may be adjusted by the user to achieve customized search purposes. For all gene models passing the filter, SPADA outputs the sequences in FASTA format and gene coordinate information in GFF format. SPADA also generates for each gene family a multiple sequence alignment including all predicted models and the family-specific consensus sequence. If a gene annotation file has been passed to the pipeline, SPADA will also report the comparison results of predicted models with existing annotation (e.g., the number of models with exactly the same exon boundaries, models with partial overlap, models in different reading frames, etc.).

### Performance evaluation

#### Compilation of the test set

We have compiled a test set of plant cysteine-rich peptides (CRPs) in two genomes: the model dicotyledon, *Arabidopsis thaliana*, and the model legume, *Medicago truncatula*. In previous work, Silverstein *et al.* have exhaustively searched and curated all 516 CRP families (CRP0000 - CRP6250) in Arabidopsis [11]. A large number of CRPs have also been identified and curated in an early release of the *Medicago* genome sequence [11]. Recently, as a collaborative effort with J. Craig Venter Institute (JCVI), we expanded this list of CRPs in *M. truncatula*. We manually inspected and curated 136 CRP families (CRP0000 - CRP1530, focusing specifically on the Defensin-Like proteins or DEFLs) in *M. truncatula*[31]. Finally, we now have a complete list of CRP members for Arabidopsis and *Medicago* (742 for Arabidopsis and 725 for *Medicago*, Additional file 1: File S1).

We collected evidence from multiple sources to validate the expression of the models in the compiled test set. On the one hand, extensive RNA-Seq data were downloaded from NCBI Sequence Read Archive [32] for both Arabidopsis and *Medicago*; on the other hand, we downloaded the AtMtDEFL microarray dateset [12,13] to find additional support for expression of these gene families. The AtMtDEFL array include probe sets for 317 Arabidopsis DEFLs, 15 Arabidopsis DEFL-related Genes (MEGs), and 684 *Medicago* DEFLs, plus additional marker genes. In total, 583 (78.6%) out of the 742 CRPs in the Arabidopsis test set and 657 (90.6%) out of the 725 *Medicago* CRPs receive support from either RNA-Seq (FPKM >1) or microarray data (Additional file 2: Table S3 and Additional file 3: Table S4). These carefully curated, high-quality CRP calls were then taken as our test set in evaluating the performance of different model prediction components in SPADA under different hmmsearch E-value cutoffs. All experimental procedures complied with the guidelines of the Institutional Biosafety Committee (IBC) at University of Minnesota (IBC code: 1301-30313H).

#### Evaluation procedure

We tested a number of popular gene prediction programs as SPADA model prediction components. In addition to the previously mentioned components, we also tested GeneID (v1.4.4) [16], GlimmerHMM (v3.0.1) [14], GeneMark (v3.9d) [15], and Augustus (v2.6.1, *de novo* mode). The "Augustus_evidence" differs from the "Augustus_de_novo" component simply by the inclusion of a "hint file" (with hit location information) fed to the program. We evaluated the pipeline performance running these components (individually or in combination) based on our curated test dataset (see Results and Discussion), and decided to use the "Augustus_evidence" and "Genewise+SplicePredictor" approaches as default components in the SPADA model prediction step.

Prediction performance was measured at two different levels: coding nucleotide sequence and exonic structure. At each level, we measured the sensitivity and specificity for each component. We first define the true positives (TP, number of coding nucleotides that are correctly predicted as coding), true negatives (TN, number of noncoding nucleotides that are correctly predicted as noncoding), false negatives (FN, number of coding nucleotides predicted as noncoding) and false negatives (FN, number of noncoding sequences predicted coding). At the nucleotide level, Sensitivity (Sn) is then defined as the proportion of coding nucleotides that have been correctly predicted as coding $\left(\text{Sn}=\frac{\text{TP}}{\text{TP}+\text{FN}}\right)$, while Specificity (Sp) is the proportion of predicted coding nucleotides that are actually coding $\left(\text{Sp}=\frac{\text{TP}}{\text{TP}+\text{FP}}\right)$[33]. At the exon level, Sn is the proportion of actual exons in the input sequence that are correctly predicted, while Sp is the proportion of all predicted exons that are correctly predicted [33]. Other measures such as Correlation Coefficient (CC) and Average Conditional Probability (ACP) were not evaluated since they require the calculation of TN nucleotides/exons, which are noncoding regions that are predicted as noncoding. Unlike a general gene-finding program that tries to predict all coding genes in a given sequence, SPADA focuses only on coding genes that are significantly similar to a given profile, while ignoring all other genes. Consequently "TN" statistics is not straightforward to evaluate in this context.

Performance evaluation was done in both *A. thaliana* and *M. truncatula*. The extracted genomic sequences were used as input sequences. We evaluated the pipeline performance using each of the "GeneID", "Augustus_de_novo", "GlimmerHMM", "GeneMark", "GeneWise+SplicePredictor", "Augustus_evidence" component (individually), as well as "SPADA" (combination of "GeneWise+SplicePredictor" and "Augustus_evidence") and "All" (combination of all 6 individual components). All programs were installed and run locally on a GNU/Linux workstation. The appropriate parameter files, model files and training directories, if available, were used to run these programs in each species, otherwise the default parameter files (which are for Arabidopsis) were used. The output of these runs were parsed to derive a unique prediction for each test sequence.

### RNA-Seq and microarray processing, data visualization

We mapped the RNA-Seq short reads (downloaded form NCBI SRA) to the reference using TopHat and summarized the results using Cufflinks [34]. Cufflinks is able to estimate the expression value at the level of transcripts. We used a cutoff of FPKM (Fragments Per Kilobase of exon model per Million mapped fragments) > 1 to determine if a model (either in the test set or in the SPADA prediction set) is expressed.

For the AtMtDEFL array, PMA (Present, Marginal and Absent) calls and normalized expression values of each probe set were obtained from the supplemental tables of two recent papers [12,13]. We mapped the probe sequences to the transcript models in the test set as well as SPADA prediction. In many cases the annotated gene boundaries are not complete and lack portions of the 3’-UTR that is prioritized in Affymetrix designs, and the probes designed in these regions would not be mapped. As a result, we require at least six probes in a probe set matching the target gene (with 23 or more identical nucleotides for each 25-mer oligo probe). Finally, the PMA calls of a probe set should be 'Present’ in at least one tissue/treatment condition to indicate expression support for the transcript model it is mapped to.

In order to visualize some of the novel SPADA predictions as compared to the original genome annotation, as well as the underlying RNA-Seq read mapping support, we loaded the data (genome sequence file, annotation GFF file, SPADA prediction GFF file, RNA-Seq mapping BAM file) into IGV (Integrative Genomics Viewer [35]), adjusted the width of each track, and made screenshots.

## Results

### Performance evaluation of SPADA on plant Cysteine Rich Peptide (CRP) families

#### SPADA performance under different search E-value thresholds

Using our manually-curated high-quality CRP test set from Arabidopsis and *Medicago*, we first evaluated the performance of SPADA under different search E-value thresholds. Generally speaking, with a loose E-value threshold (e.g., 0.1), SPADA is able to predict almost all true models (i.e., achieving high sensitivity) while making many false predictions (i.e., specificity is low) (Additional file 4: Figure S1). By setting the search threshold to a more stringent value, SPADA avoids making most of the false predictions, but also loses a small number of true models. In an effort to optimize search sensitivity (the ability to detect all true gene models) and specificity (prevent detection of spurious false models; refer to the "Method / Performance evaluation / Evaluation procedure" section for a formal definition of Sensitivity and Specificity), we set the default search E-value threshold to 0.001. Users can also change the default E-value threshold to build custom searches (e.g., a very sensitive search using E-value cutoff of 1 to identify all potential hits).

#### Performance comparison of different gene prediction components

**Figure 2:**
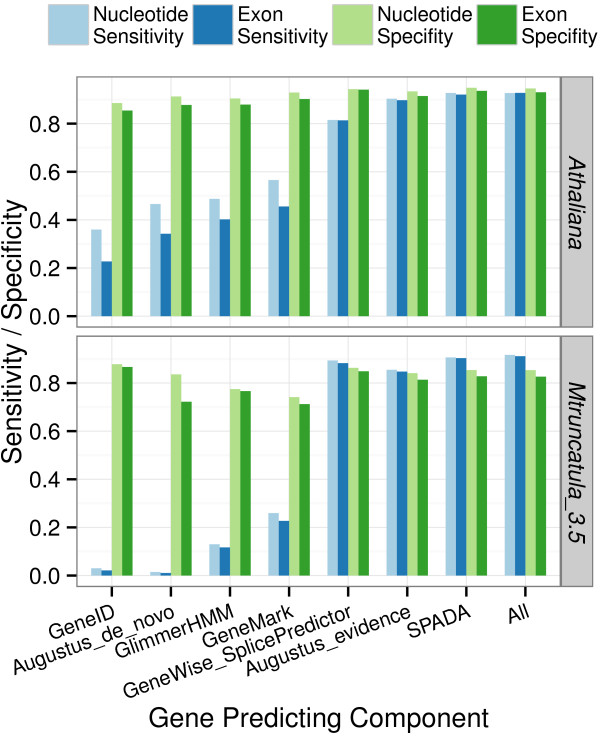
**Performance comparison of different gene prediction components.** Search E-value threshold is set to 0.001 by default.

We then compared the performance of SPADA running different model prediction components: GeneID, Augustus ("*de novo*" mode as well as "evidence" mode), GlimerHMM, GeneMark, GeneWise+SplicePredictor as well as "SPADA" (combination of "Augustus_evidence" and "GeneWise+SplicePredictor") and "All" (combination of all 6 individual components) (Figure 2, Additional file 4: Figure S1). The high specificities observed in all components are likely due to the model evaluation and selection step, where most false models are filtered. Prediction sensitivities, on the other hand, show substantial differences among components. In both genomes tested, "Augustus_evidence" and "GeneWise+SplicePredictor" gave the highest sensitivities among the six individual components. The default SPADA pipeline (denoted as "SPADA" in the figure) runs these two components and achieved even higher sensitivity. On the other hand, running all six individual components (denoted as "All" in the figure) gives the highest sensitivity, suggesting that search accuracy can still be improved by including more heterogeneous prediction programs in the pipeline. However, the gain in sensitivity offered by running all six components is marginal compared to running just two of them ("Augustus_evidence" and "GeneWise+SplicePredictor"), suggesting that a plateau in search accuracy could soon be reached and adding more prediction programs in the pipeline may not help much.

These results are expected as SPADA does not work as a general gene finding program but instead focuses on particular classes of genes with known profiles. Small genes are typically difficult to predict and often missed by genome annotation pipeline due to the intrinsic properties of many automatic gene finding algorithms [36]. In our test with GeneID, Augustus and GlimmerHMM against the *Medicago* genome, the Arabidopsis training matrix was used since a *Medicago* specific one is not yet available. This explains to a large extent the extremely low sensitivity performance for these three programs in *Medicago*. Search specificities were generally quite high and did not vary much among different programs or genomes tested, indicating the relatively stringent search E-value (0.001) in effect allows few false positives.

### Cysteine-rich peptides predicted by SPADA in Arabidopsis and *Medicago*

Using the default search E-value threshold and model prediction components, SPADA predicts 745 CRPs in Arabidopsis and 1170 (747 for CRP0000-CRP1530) in *Medicago* (Table 1, Additional file 5: Table S1, Additional file 6: Table S2, Additional file 7: Figure S2, Additional file 8: Figure S3 and Additional file 9: File S2). These numbers are generally consistent with our manually curated CRP test sets (742 for *Arabidopsis*) and 725 for *Medicago*[11], with a sensitivity of 91%–93% and specificity of 85%–95% at the nucleotide level (Additional file 4: Figure S1). Members within a sub-class typically show a conserved signal peptide and cysteine configuration (Additional file 10: Figure S4 for example). We also checked the expression of these predictions using publicly available RNA-Seq data from NCBI: 570 (76.5%) out of the 745 Arabidopsis CRPs and 947 (80.9%) out of the 1170 *Medicago* CRPs receive either RNA-Seq or AtMtDEFL array expression support (Additional file 11: Table S5 and Additional file 12: Table S6). It should be noted that SPADA makes no attempt to predict pseudogenes as it filters out hits with in-frame stop codons. However, some pseudogenes with premature stop codons might still be predicted by SPADA as valid gene models if the in-frame part shows significant (though incomplete) similarity to the search HMM. This in part explains the higher number of SPADA predictions (747 for CRP0000-CRP1530) in *Medicago* than our test set (725) since pseudogenes were manually removed to obtain the test set.

**Table 1:** **Cysteine-Rich Peptides (CRPs) predicted in *A. thaliana * and** ***M. truncatula***

		***A. thaliana***	***M. truncatula***
Defensin related	CRP0000-CRP0260,etc.	56	43
LCR/BET1 related	CRP0280-CRP0810,etc.	162	110
SCR related	CRP0830-CRP0880	32	6
Metallocarboxypeptidase inhibitor	CRP1004-CRP1030	0	1
CCP related	CRP1040-CRP1120	19	4
Nodule Cysteine-Rich peptide	CRP1130-CRP1530	3	583
Ripening related protein	CRP1600-CRP1605	0	21
Novel family	CRP1620,CRP2800,etc.	14	15
Miscellaneous	CRP1640-CRP1660,etc.	16	48
Rapid Alkalinization Factor	CRP1700-CRP2120	38	36
Thionin related	CRP2200-CRP2610	66	23
Root cap/late embryogenesis	CRP2820-CRP2850	5	7
Antimicrobial peptide MBP-1	CRP2900-CRP3000	1	2
Bowman Birk inhibitor	CRP3100-CRP3190	0	16
Pollen Ole e I	CRP3300-CRP3510	34	44
ECA1 gametogenesis related	CRP3600-CRP3740	124	17
Lipid transfer protein	CRP3800-CRP4962	127	127
2S Albumin	CRP4970-CRP5080	5	3
Glutenin/Giadin/Prolamin	CRP5090-CRP5270	0	0
Maternally-expressed gene/Ae1	CRP5300-CRP5520	20	2
Proteinase inhibitor II	CRP5545-CRP5600	6	2
Chitinase/Hevein	CRP5610-CRP5820	10	15
Kunitz type inhibitor	CRP6010-CRP6180	7	45
Total		745	1170

The default E-value threshold of 0.001 is a compromise between sensitivity and specificity that generally works well for both organisms. For the purpose of identifying all potential small coding genes, a search with high sensitivity should be performed since it allows the user to see all potential hits and then determine for him/herself the boundary between false predictions and true predictions based on search scores. The users can then set the cutoff threshold empirically and select genes for experimental verification on their own. Thus, we also report here two CRP prediction sets by running SPADA using E-value threshold of 1 (Additional file 13: Table S7 and Additional file 14: Table S8). In practice, users are encouraged to change the default E-value threshold to build custom searches.

**Figure 3:**
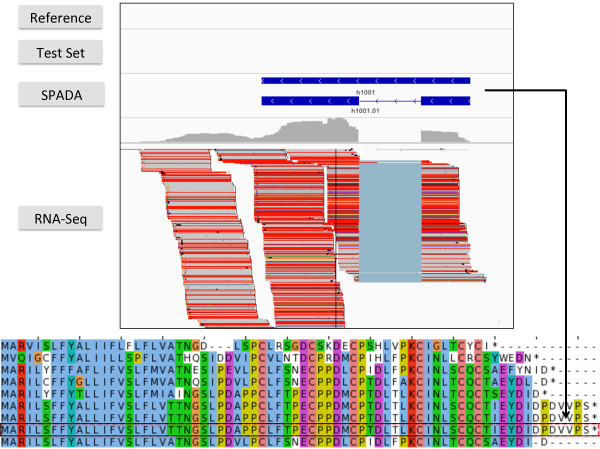
**A novel gene model predicted by SPADA is missed by the current *Medicago * annotation.** A *Medicago* NCR (h1001.01) shown in IGV (above figure) and subgroup alignment of CRP1180 (below figure, h1001.01 shaded).

According to the latest versions of genome annotation for Arabidopsis and *Medicago*, about 5% to 15% of SPADA predictions fall completely into intergenic regions (i.e., are un-annotated, Table 2). Through manual inspection of these models, we found that some of the unannotated models turn out to be ORFs with premature stop codons (i.e., pseudogenes), while others had quite significant hmmsearch E-value and complete ORFs. In addition, some predicted models receive expression support from either existing EST sequence or RNA-Seq data. An example is shown in Figure 3 where the predicted CRP model is supported by RNA-Seq mapping, fits well in the family-specific alignment, but was missed by the genome annotation (*Medicago* genome annotation version 3.5) as well as by our test set. While such cases are infrequent (e.g., only 10 in Arabidopsis), we speculate the specificity of SPADA is likely to be underestimated.

**Table 2:** Novel CRP models identified by SPADA determined by manual inspection

	***A. thaliana***	***M. truncatula***
Total predictions	745	1170
Number of unannotated predictions^*a*^	5	125
Number of novel models^*b*^	3 (60%)	77 (62%)

^*a*^An unannotated prediction is a gene model predicted by SPADA but missed by current genome annotation.

^*b*^Novel models are unannotated predictions that are manually inspected to be true members of the family with evidence from family-specific alignment and/or RNA-Seq evidence.

We then performed manual inspection on these unannotated CRP models and tried to determine whether the calls are truly bad predictions (e.g., pseudogenes with pre-mature stop codons) or valid members of the family missed by current genome annotation (criteria being that the predicted model fits well in the family-specific alignment and has either RNA-Seq or Affymetrix expression support). The number of "novel" CRPs discovered in this fashion, is given in Table 2 (Additional file 15: File S3). SPADA was able to identify 77 novel CRPs in *Medicago* that were missed by current genome annotation pipeline. The actual number of new CRPs in *Medicago* will be even higher since we only evaluated a subset of all CRP groups (CRP0000-CRP1530). This result is not unexpected given that the *Medicago* genome was released only recently and resources and efforts put into the genome annotation pipeline have been limited. On the other hand, only 3 novel CRPs were found in Arabidopsis, suggesting a relatively higher quality of gene calls in this extensively studied model organism.

Through examination of the novel CRPs, we also noted that while some of the novel hits have a very significant hmmsearch E-value (e.g., 10^-12^), most have moderate E-values (e.g., 10^-4^-10^-7^), suggesting that their sequence similarity to the input HMM profile is limited. While the input profile alignments were manually built and may not be exhaustive in capturing all groups of CRPs, we speculate that some of these novel CRPs might form new clades that define novel profile alignments, separate from the original alignment. Consequently, a new round of genome scans using these novel profiles has the potential for capturing even more members that have been missed in the previous search.

### Case study: the S-Protein Homologue (SPH) family in Arabidopsis

**Figure 4:**
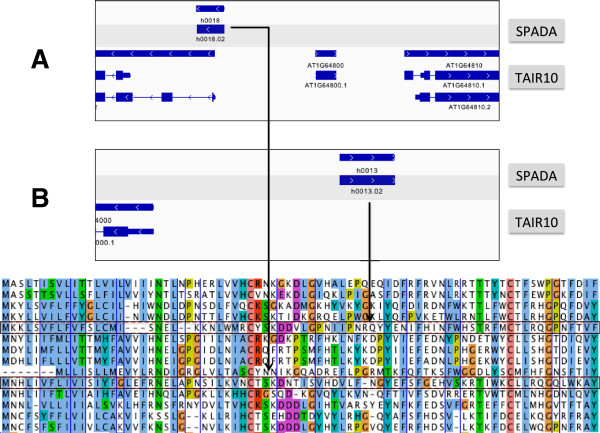
**SPADA detects mis-annotated and novel SPH peptides in TAIR10.** **(A)** SPADA detects an SPH peptide (h0018.02) that is mis-annotated in TAIR10; **(B)** SPADA detects a novel SPH peptide (h0013.02) not present in TAIR10. Multiple sequence alignment of selected SPH peptides are shown below with h0018.02 and h0013.02 shaded.

In addition to plant CRPs, SPADA is readily generalizable to other classes of putative secreted peptides by substituting an appropriate sets of HMMs in place of CRP HMMs. Here, we used the SPH peptides (S-Protein Homologue) [37] as an example. A seed alignment including 45 plant self-incompatibility protein S1 sequences (PF05938) was obtained from the Pfam database. An HMM profile was built from this alignment and then used as input to scan the Arabidopsis genome (TAIR10 [38]) by running SPADA. SPADA predicted 92 SPH peptides in total (Additional file 16: Table S9 and Additional file 17: File S4). Forty-five (45) of these predictions are identical with TAIR10 annotation. Seventeen have minor discrepancies with TAIR10 gene models (coding regions all in the same reading frame but have a boundary conflict of less than 15 amino acids, probably resulting from different start codons or alternative splice sites). Nine are in major conflict with existing gene models (coding regions in different reading frames or having serious boundary conflict). We also discovered 21 new SPHs not present in TAIR10. Through manual inspection of gene models and sequence alignments with other family members, we found 3 out of the 15 major conflicts reflect an error in TAIR10 (Figure 4A gives an example), while 19 out of the 21 models absent from TAIR10 are true members of the SPH family, missed by the current genome annotation (Figure 4B shows an example). Thus, we demonstrate that SPADA accurately detects other classes of secreted peptides given a well-constructed profile alignment.

### Case study: a fungal cyclic peptide family in *Amanita bisporigera*

In order to assess whether SPADA could be useful in searches for families of small non-secreted peptides outside the plant kingdom, we also examined the fungal cyclic peptides of Amanita mushrooms [39]. This family includes the amatoxins and phallotoxins, such as *α*-amanitin and phalloidin, respectively, which are synthesized as proproteins of 34-35 amino acids. We began by creating a multiple sequence alignment via ClustalO of reported proproteins [39] and executed SPADA as usual with the signal peptide filter turned off, using Arabidopsis as the training model for Augustus in searching the low-coverage genome contigs of *Amanita bisporigera*. As a negative control, we scanned the genome of *Amanita thiersii*, which is non-toxic and not known to produce this class of toxins.

**Figure 5:**
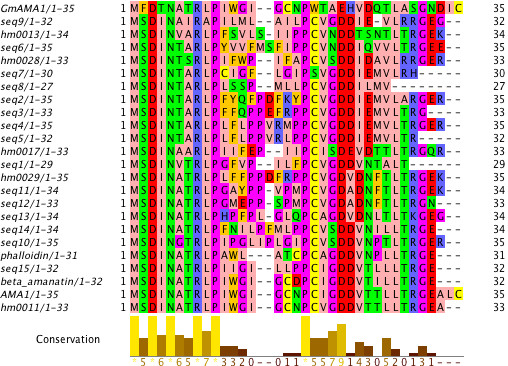
**Multiple sequence alignment of Amanita toxin proproteins.** Sequences identified by SPADA are labeled as "hm****". All remaining sequences were obtained from Hallen *et al.*[39], and were included in the initial alignment used as input for SPADA.

SPADA identified five new peptides in the incomplete *A. bisporigera* genome with strong homology (2.4 × 10^-17^ <E < 5.8 × 10^-8^) that fit well with the alignment of known proproteins (Figure 5). One additional hit ran off the end of the contig, producing the incomplete propeptide "MSDTNVMRLPFTTP". No additional predictions were made by SPADA with E < 0.01 beyond sequences that were already included in the original alignment. Further, SPADA did not identify any hits when scanning the genome of *A. thiersii*, as would be expected from that organism’s non-toxic nature.

## Discussion

### Homology-based gene prediction

Unlike general-purpose gene predicting programs, SPADA works as a family-based gene finder. The major difference between SPADA and general gene predicting programs is that it incorporates prior information from the family profile in the prediction process. SPADA takes advantage of generic gene prediction programs, but goes a step further by suggesting where to look for family members. Through scanning the target genome using pre-built family-specific alignments, SPADA identifies and builds "extented hits" that serve as the backbone of the underlying exonic structure. This location information greatly improves prediction accuracy, as shown by the different performances of "Augustus_*de_novo*" and "Augustus_evidence" components in Figure 2. Among the six individual predicting components, the four that do not require additional information and make *de novo* predictions all yield low sensitivities. The other two approaches, "Augustus_evidence" and "GeneWise+SplicePredictor", make use of the location information and are able to predict most of the true positives.

Family-based gene prediction was first introduced in the AUGUSTUS package as the AUGUSTUS-PPX (Protein Profile eXtension) module [17]. Although AUGUSTUS-PPX was shown to be more sensitive and accurate in predicting long, multi-exon gene family members than the standard AUGUSTUS algorithm, its approach is not suitable for small, divergent peptide families such as CRPs, SPHs or Amanita toxin-like peptides examined here. Rather than using the entire protein family alignment profile as input, AUGUSTUS-PPX makes use of conserved, ungapped blocks from the alignment to make a profile. This enables the algorithm to identify core match regions in the genome sequence which together act as a scaffold in the gene prediction. A modification of the standard AUGUSTUS gene-centric HMM is then used to fill in the pieces between scaffold elements with splice elements and other signals, ultimately emitting the most probable full gene structure. While this approach works well for families of typical genes with large numbers of conserved elements, it completely breaks down when applied to small, divergent peptide families like the CRPs, as these families tend to contain no conserved, ungapped regions of appreciable size to seed the initial scaffold. Indeed, when we applied AUGUSTUS-PPX to the CRPs we observed no improvement over "Augustus_*de_novo*".

### Improving prediction accuracy by model evaluation

The default SPADA pipeline (running the "Augustus_evidence" and "GeneWise+SplicePredictor" components) achieves even higher sensitivity than the two individual components. This owes to the model evaluation & selection step. For each HMM hit, SPADA collects all candidate gene models built by its model predicting components, and in the model evaluation step, picks a best candidate model based on multiple evaluation statistics. True family members will probably get a high-scoring gene model, while most false positive hits will have no qualifying or only low-scoring gene models built. High-scoring gene models that passed the filter are more likely to be true models since they are the ones that best fit the family-specific alignment.

### Pseudogenes and gene models without expression evidence may still have significant value

SPADA identifies paralogous gene family members throughout the genome. Many of these predictions currently lack expression evidence and some of the gene predictions have premature stop codons suggesting they may be psuedogenes. Nonetheless, it is important to identify all gene family members, regardless of their expression and pseudogene status, especially in evolutionarily dynamic gene families,. The semi-automated approach that inspired SPADA’s development identified hundreds of defensin-like genes in Arabidopsis which, at the time, had no expression evidence [40]. Later, these genes turned out to be highly specifically expressed in reproductive tissues not previously examined with earlier genome-wide expression approaches [41]. Moreover, one must also be careful not to discard pseudogene predictions that are highly similar to other family members. A gene that appears as a pseudogene in the reference sequenced accession of a species may indeed be fully intact in other accessions, as observed among the defensin-like pollen-tube attractant, AtLUREs [42]. In their study, Takeuchi and Higashiyama observed half a dozen AtLUREs with disabling mutations in non-reference accessions, as well as putative functional and intact forms of AtLUREs 1.5 and 1.6, which are pseudogenes in the reference Col-0 genotype [42].

### Improving SSP annotation in current plant SSP databases

Previous work has sought to exhaustively identify small secreted peptides (SSP) in Arabidopsis [4], rice [7] and *Populus deltoides*[6]. These earlier studies only scanned short ORFs (25-250 amino acids) in translated genome sequence, though Pan *et al.*[7] did include multiple-exon gene predictions from *ab initio* gene predition programs such as Fgenesh and Augustus. However, with a primary focus on detecting all small secreted peptides, these studies did not utilize protein family information in the model building process since secreted peptides are so diverse. The Arabidopsis Unannotated Secreted Peptide Database (AUSPD) only contains one-exon ORF predictions, and thus mis-annonates most (if not all) two-exon secreted peptides (Additional file 18: Figure S5 for example). The OrysPSSP database (comparative Platform for Small Secreted Proteins from rice from rice and other plants) does contain multi-exon models predicted by Fgenesh (0.72%) and Augustus (1.16%) in addition to single-exon ORFs [7]. However, since no prior information is incorporated into predictions by these *ab initio* gene predicting programs, multi-exon models in OrysPSSP are frequently in conflict with the true rice CRPs (Additional file 19: Figure S6 for example). As a result, while most single-exon peptides in Arabidopsis and rice are captured in AUSPD and OrysPSSP respectively, a large portion of the two-exon and multi-exon genes (such as CRP0000-CRP1530) are clearly under-represented in these two databases. SPADA, on the other hand, used additional gene structure information obtained in the motif mining step and was able to correctly predict most of the CRP models (Additional file 20: Table S10).

### Complementarity of SPADA to generic gene prediction programs

SPADA is not designed to identify all genes in a genome. However, its applicability to new annotation projects steadily will increase due to the marked growth of protein sequence family signatures and alignments. InterPro release 43.0 contains 16,652 protein family signatures. In the last 3 years, the number of families characterized by InterPro has increased by 24%, compared with a 38% increase in the 3 years prior to that [9]. (Release 29.0 from October 2010 had 13,382 family entries; Release 16.1 from October 2007 had 9,729 entries.) It should be noted that SPADA is unlikely to perform well with genes that have large numbers of exons due to the combinatoric explosion of potential splice donor and acceptor pair combinations to evaluate. For longer multi-exon gene families, AUGUSTUS-PPX should be used. Still, SPADA has been shown here to be extremely effective in predicting families of one- and two- exon genes often missed or excluded by standard gene prediction algorithms [3,4]. Hence, it is anticipated that gene annotation pipelines would be improved by routinely running SPADA to pick up small genes in addition to the standard generic gene prediction algorithms (e.g., Augustus) for larger genes.

### Impact of better gene prediction algorithms on plant genomics

As sequencing costs have come down, there has been a commensurate expansion in the sequencing of multiple plant genomes within each species. Moreover, Genome Wide Association Studies (GWAS) are now routinely carried out in these populations. Gene annotation cannot be simply transferred across members of a species due to the myriad of SNPs and indels that alter gene structures. Gan *et al.* estimated that gene structural changes occurred in more than 30% of genes among the 18 Arabidopsis accessions they resequenced and assembled [43]. Further, GWAS studies have repeatedly implicated unannotated intergenic regions as having the most significant association with important agronomic traits [44,45]. While it is likely that many of these GWAS peaks identify non-coding RNAs or regions in strong linkage disequlibrium with causative variants, we suspect that many of these sites may actually mark members of as yet unannotated families of small genes. Indeed, in our own GWA studies [46], many peaks turned out to coincide with NCR or other CRP family members that prior to our intensive family-based annotation studies had been un-annotated in *Medicago*.

### Discovery of genes resembling Nodule-Cysteine-Rich (NCR) peptides in Arabidopsis

In striking contrast to Arabidopsis, the *Medicago* genome harbors a huge number (583 versus 3) of Nodule Cysteine-Rich peptides (NCRs, CRP1130-CRP1530) – Defensin-Like proteins with nodule-specific expression (Table 1). These NCRs are unique to *Medicago* (specifically, legumes in the Inverted Repeat-Lacking Clade) [47] and have recently been shown to play vital roles in the communication between *Medicago* and symbiotic rhizobia [48,49].

Surprisingly, three CRPs were found in the Arabidopsis genome falling into the nodule-specific sub-families (CRP1130-CRP1530, or NCRs). Previously, NCRs were thought to be unique to *Medicago* and other IRLC legumes, playing a vital role in the legume-rhizobia symbiotic interaction [50]. Looking closely at the sequence alignments (Additional file 21: Figure S7), these "Arabidopsis NCRs" have all the conserved cysteine residues in the expected configuration, while also exhibiting substantial divergence from *Medicago* NCRs - and forming a separate Arabidopsis-specific clade. Furthermore, only one Arabidopsis NCR is predicted in each sub-class. It is possible, therefore, that these "Arabidopsis NCRs" are descendants from the most recent common ancestral genes that later evolved into *Medicago* NCRs. After the Arabidopsis-*Medicago* divergence, these ancient NCRs could have become increasingly divergent in the legume (*Medicago*) clade, eventually gaining new functions in nodule development and symbiosis, possibly through neo-functionalization, conferring a selective advantage and thus increasing rapidly in copy number through gene duplication.

### Limitations of the SPADA pipeline

Because the model prediction step in the pipeline is not optimized for multi-exon gene models nor the extremely large introns present in animal genomes, we do not yet recommend SPADA to identify small peptides in animals (especially mammals). Also, SPADA is not expected to work well with bacterial genomes due to the absence of introns in their gene models. However, we speculate this pipeline will work well with organisms such as yeast, oomycete and fungi, since they have similar gene structures to plants [51,52]. In fact, it was recently found that oomycetes and fungi genomes encode large number of secreted effectors as a result of the evolutionary "arms-race" between pathogen and host [53,54]. Potentially, SPADA will be useful in effector discovery in these pathogen genomes given that a growing number of informative family alignments are becoming available.

### The SPADA pipeline is useful beyond secreted peptides and outside plants

Although SPADA was initially designed to target secreted peptide families in plants, it can be used on non-secreted peptide families, especially in fungal systems. In the Results section, we tested SPADA using draft genome contigs of the mushroom *A. bisporigera* in search of additional members of a class of potent liver toxin pro-peptides characterized in earlier work [39]. Roughly 20 pro-peptides belonging to this family had been cloned and sequenced, with only about a dozen present among the draft genome sequence contigs. SPADA identified 5 new family members with convincing alignments and significant E-values (2.4 × 10^-17^ < E < 5.8 × 10^-8^). Three of these were in contigs long enough that extensive homology in the 3’-UTR region characteristic of the family could be observed. The contigs of the remaining two hits ended shortly after the coding sequence preventing 3’-UTR homology characteristics from being confirmed. When the same input HMM constructed from the known 20 pro-peptides [39] was used to scan against a related mushroom *Amanita thiersii* that is known not to produce toxic peptides, SPADA did not identify any candidate genes with E < 0.01.

## Conclusions

SPADA is a homology-based gene prediction program to accurately identify and predict the gene structure for short peptides with one to a few exons. SPADA works well on small plant peptides such as the cysteine-rich peptide families. SPADA gives much more accurate and precise gene calls than traditional *ab initio* gene finding programs in tested genomes. Running SPADA on less well-annotated plant genomes (e.g., *Medicago*) reveals numerous mis-annotated and unannotated CRPs in the current genome annotation. Predictions made by SPADA constitute the most complete set of plant cysteine-rich peptides, and in this regard, will provide an invaluable resource for the research of small, secreted peptides in plants. The systematic application of SPADA to other classes of small peptides by communities will greatly improve the genome annotation of different protein families in public genome databases.

## Availability and requirements

**Project name:** Small Peptide Alignment Discovery Application

**Project home page:**https://github.com/orionzhou/SPADA

**Operating system(s):** Linux

**Programming language:** Perl

**Other requirements:** SPADA is a perl-based pipeline that internally runs HMMer, Augustus, Genewise, SplicePredictor and a number of custom scripts. Instructions for installation and running are available at https://github.com/orionzhou/SPADA/wiki.

**License:** Apache License, Version 2.0

**Any restrictions to use by non-academics:** None

## Abbreviations

ORF: Open reading frame; CRP: Cysteine-rich peptide; DEFL: Defensin-like protein; NCR: Nodule cysteine-rich peptide; PMA: Present, marginal and absent; GWAS: Genome wide association study; SPH: S-protein homologue; HMM: Hidden Markov model; GFF: General feature format; MSA: Multiple sequence alignment; IRLC: Inverted repeat-lacking clade.

## Competing interests

The authors declare that they have no competing interests.

## Authors’ contributions

PZ: wrote software, significantly contributed to the concept and design of the software, carried out primary analysis, drafted and revised the manuscript; KATS: conceived of the idea for the software, extensively tested the software and contributed to its development, reviewed and edited the manuscript; LG: extensively tested the software and contributed to its development; JDW: provided access to the *A. bisporigera* draft genome, carefully examined homology in the 3’-UTR region of the 5 putative hits in that genome, and reviewed and edited the manuscript; SN: extensively tested the software and contributed to its development, reviewed and edited the manuscript; JG: extensively tested the software and contributed to its development; NDY: significantly contributed to the concept of the software, reviewed and edited the manuscript. All authors read and approved the final manuscript.

## Supplementary Material

Additional file 1 **File S1.** Manually curated CRPs (test set) in *A. thaliana* and *M. truncatula* (in GFF3 format). Click here for file

Additional file 2 **Table S3.** Expression support of the Arabidopsis CRP test set. Click here for file

Additional file 3 **Table S4.** Expression support of the *Medicago* CRP test set. Click here for file

Additional file 4 **Figure S1.** Performance comparison of five gene prediction components under different search E-value thresholds. Click here for file

Additional file 5 **Table S1.** CRPs predicted by SPADA in *A. thaliana* using E-value threshold of 0.001. Click here for file

Additional file 6 **Table S2.** CRPs predicted by SPADA in *M. truncatula* using E-value threshold of 0.001. Click here for file

Additional file 7 **Figure S2.** Genome distribution of CRPs predicted in *Arabidopsis thaliana*. Click here for file

Additional file 8 **Figure S3.** Genome distribution of CRPs predicted in *Medicago truncatula*. Click here for file

Additional file 9 **File S2.** CRP predictions made by SPADA in *A. thaliana* and *M. truncatula* using search E-value threshold of 0.001 (in GFF3 format). Click here for file

Additional file 10 **Figure S4.** Multiple sequence alignments of *Medicago* CRP sub-families CRP0000 and CRP1400. Click here for file

Additional file 11 **Table S5.** Expression support of the CRPs predicted by SPADA in A. thaliana. Click here for file

Additional file 12 **Table S6.** Expression support of the CRPs predicted by SPADA in *M. truncatula*. Click here for file

Additional file 13 **Table S7.** CRPs predicted by SPADA in *A. thaliana* using E-value threshold of 1. Click here for file

Additional file 14 **Table S8.** CRPs predicted by SPADA in *M. truncatula* using E-value threshold of 1. Click here for file

Additional file 15 **File S3.** Novel CRP predictions made by SPADA in *A. thaliana* and *M. truncatula* as determined by manual inspection (in GFF3 format). Click here for file

Additional file 16 **Table S9.** SPH peptides predicted by SPADA in *A. thaliana*. Click here for file

Additional file 17 **File S4.** SPH predictions made by SPADA in Arabidopsis using search E-value threshold of 0.001 (in GFF3 format). Click here for file

Additional file 18 **Figure S5.** A typical Arabidopsis CRP mis-annonated in Arabidopsis Unannotated Secreted Peptide Database (AUSPD). Click here for file

Additional file 19 **Figure S6.** A typical rice CRP mis-annonated in OrysPSSP. Click here for file

Additional file 20 **Table S10.** Evaluation of SPADA, AUSPD and OrysPSSP using the manually-curated test set. Click here for file

Additional file 21 **Figure S7.** Sub-class alignments of three Arabidopsis NCRs with *Medicago* NCRs. In each alignment the first sequence comes from Arabidopsis and the rest all come from *Medicago*. Click here for file
