# Supplementary figures and images for: Detecting small plant peptides using SPADA (Small Peptide Alignment Discovery Application)
Source: BMC Bioinformatics. 2013 Nov 20;14:335. doi: 10.1186/1471-2105-14-335 (PMC3924332; doi:10.1186/1471-2105-14-335)

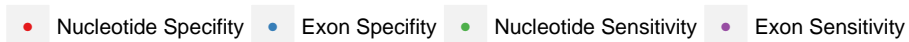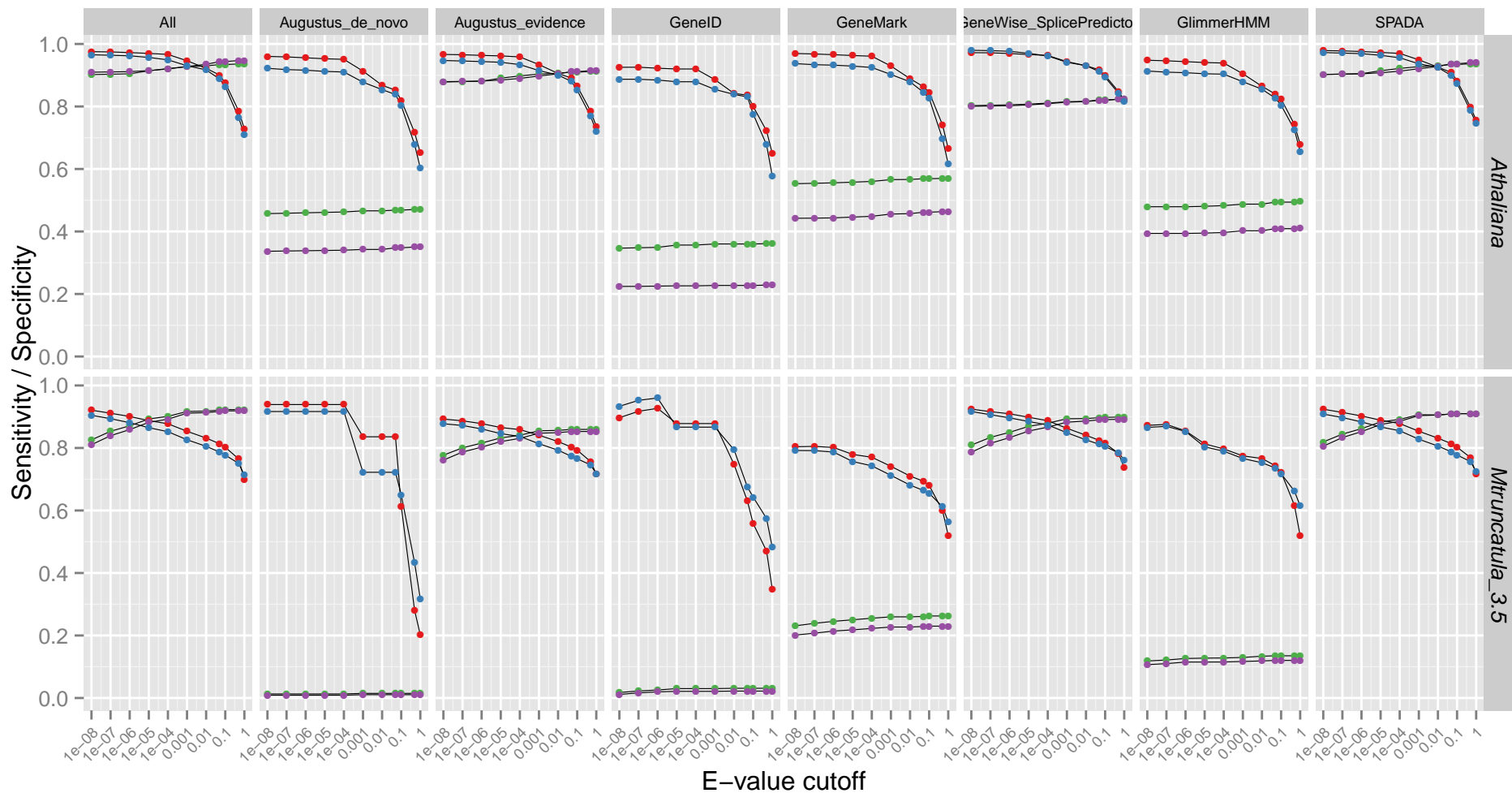

Supplement: Additional file 4 — Figure S1. Performance comparison of five gene prediction components under different search E-value thresholds. [file 1471-2105-14-335-S4.pdf]

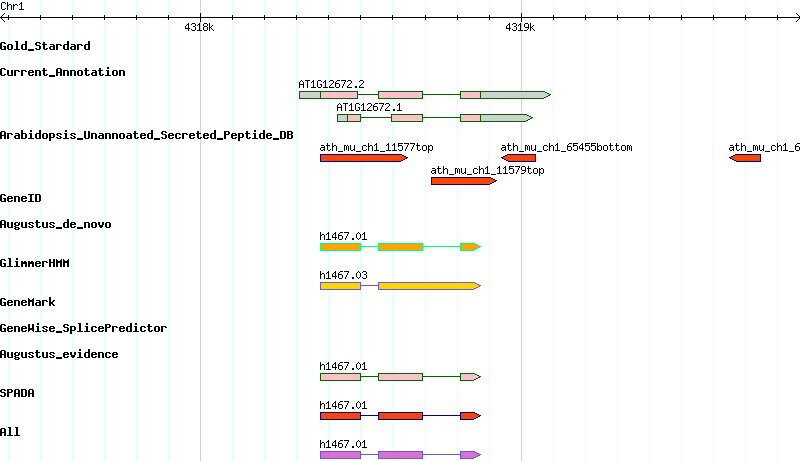

Supplement: Additional file 18 — Figure S5. A typical Arabidopsis CRP mis-annonated in Arabidopsis Unannotated Secreted Peptide Database (AUSPD). [file 1471-2105-14-335-S18.png]

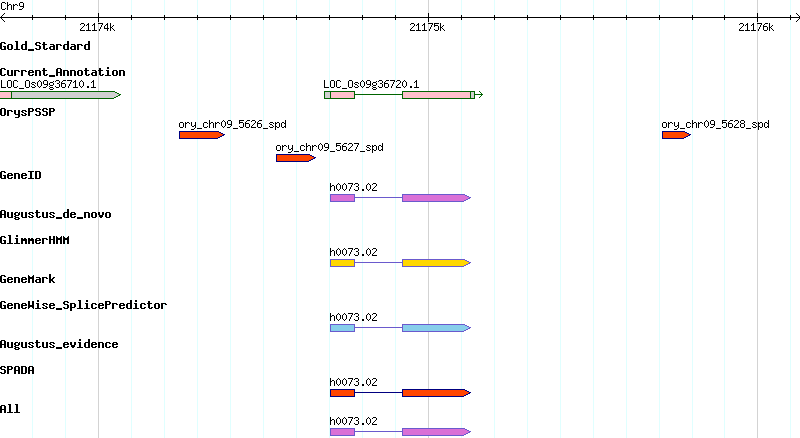

Supplement: Additional file 19 — Figure S6. A typical rice CRP mis-annonated in OrysPSSP. [file 1471-2105-14-335-S19.png]
