# Supplementary material for: Detecting small plant peptides using SPADA (Small Peptide Alignment Discovery Application)
Source: BMC Bioinformatics. 2013 Nov 20;14:335. doi: 10.1186/1471-2105-14-335 (PMC3924332; doi:10.1186/1471-2105-14-335)
Supplement: Additional file 8 — Figure S3. Genome distribution of CRPs predicted in Medicago truncatula. [file 1471-2105-14-335-S8.pdf]

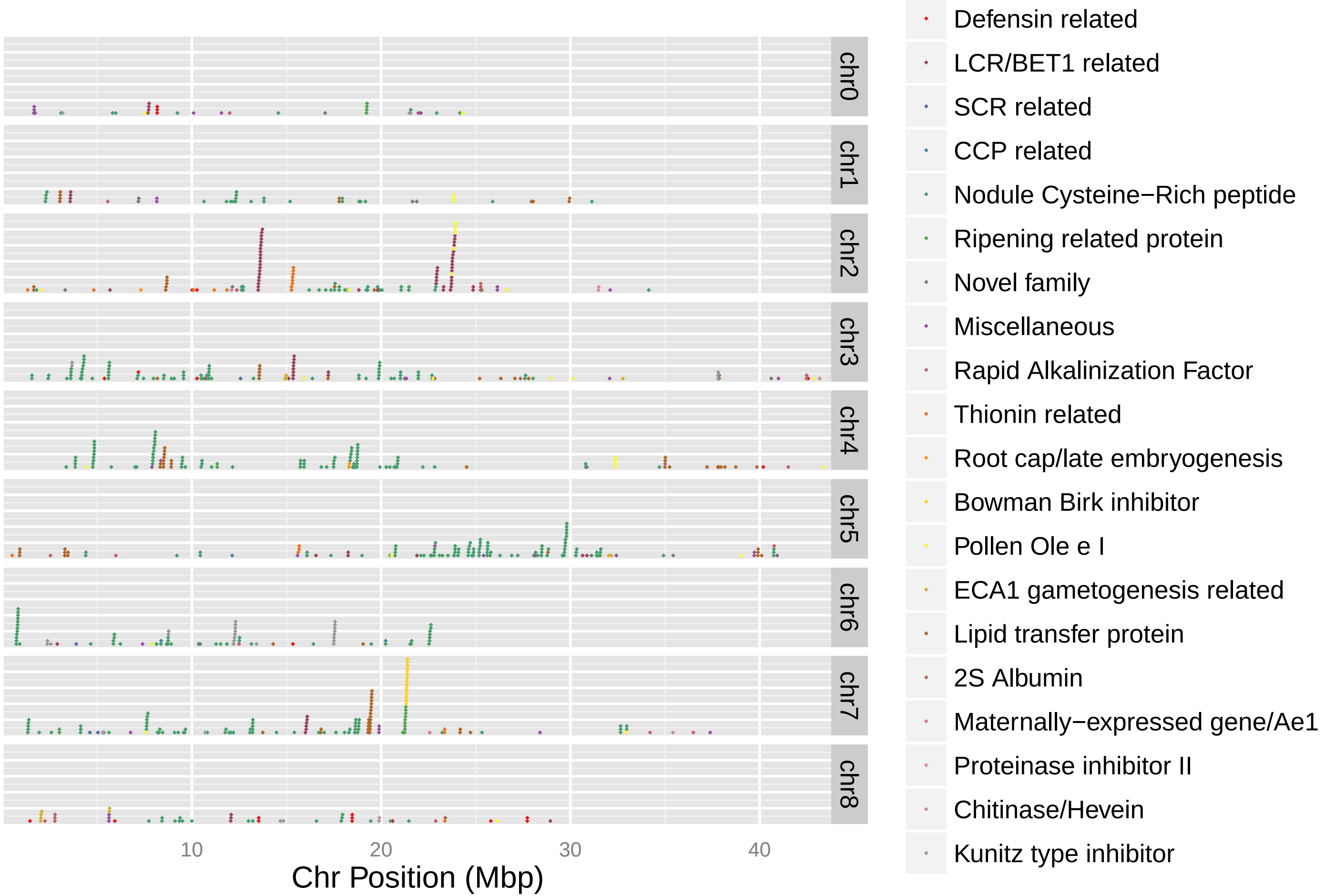
chr0
chr1
chr2
chr3
chr4
chr5
chr6
chr7
chr8
10
20
30
40
Chr Position (Mbp)
Defensin related
LCR/BET1 related
SCR related
CCP related
Nodule Cysteine−Rich peptide
Ripening related protein
Novel family
Miscellaneous
Rapid Alkalinization Factor
Thionin related
Root cap/late embryogenesis
Bowman Birk inhibitor
Pollen Ole e I
ECA1 gametogenesis related
Lipid transfer protein
2S Albumin
Maternally−expressed gene/Ae1
Proteinase inhibitor II
Chitinase/Hevein
Kunitz type inhibitor
