# Supplementary material for: Detecting small plant peptides using SPADA (Small Peptide Alignment Discovery Application)
Source: BMC Bioinformatics. 2013 Nov 20;14:335. doi: 10.1186/1471-2105-14-335 (PMC3924332; doi:10.1186/1471-2105-14-335)

CRP0000 (classic defensin)

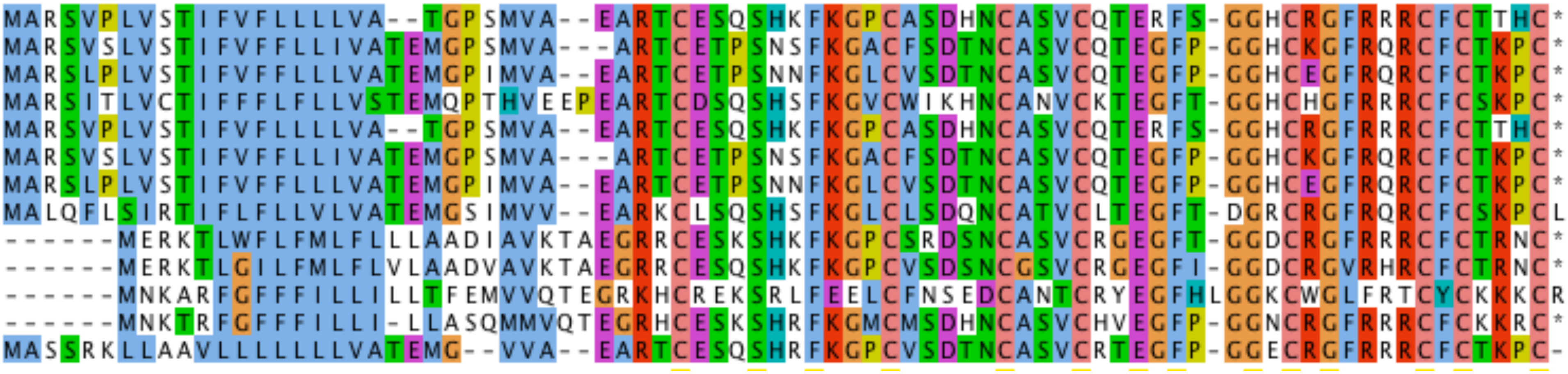

CRP1400 (nodule-specific defensin-like peptide)

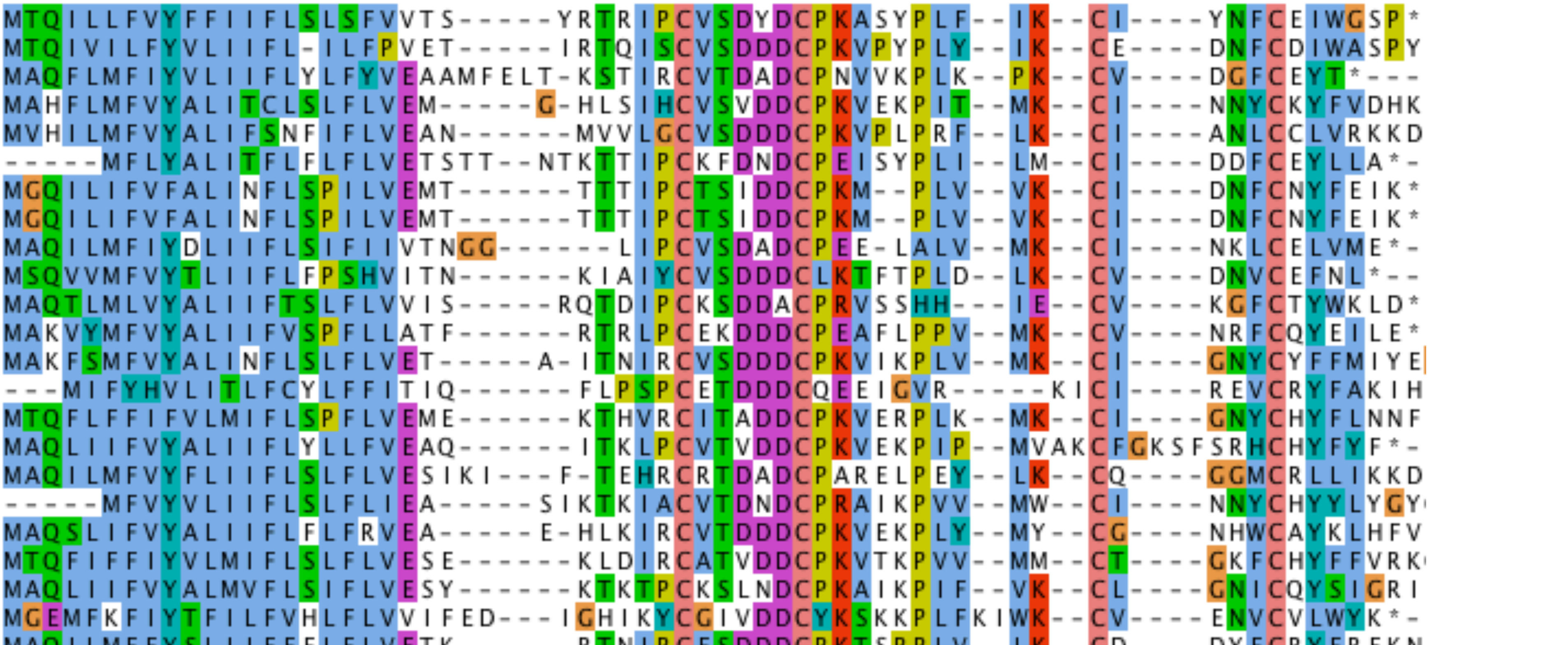

Supplement: Additional file 10 — Figure S4. Multiple sequence alignments of Medicago CRP sub-families CRP0000 and CRP1400. [file 1471-2105-14-335-S10.pdf]
