# Supplementary material for: Detecting small plant peptides using SPADA (Small Peptide Alignment Discovery Application)
Source: BMC Bioinformatics. 2013 Nov 20;14:335. doi: 10.1186/1471-2105-14-335 (PMC3924332; doi:10.1186/1471-2105-14-335)

CRP1280

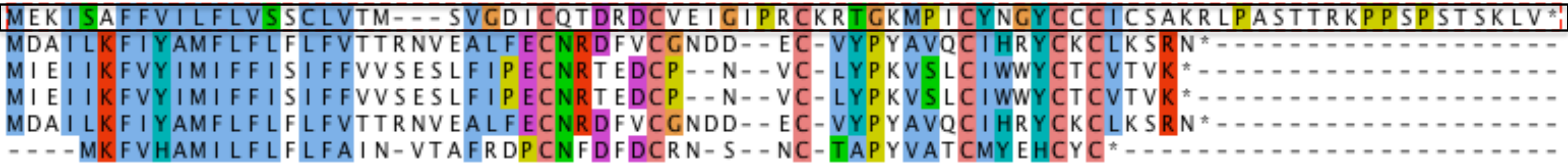

CRP1300

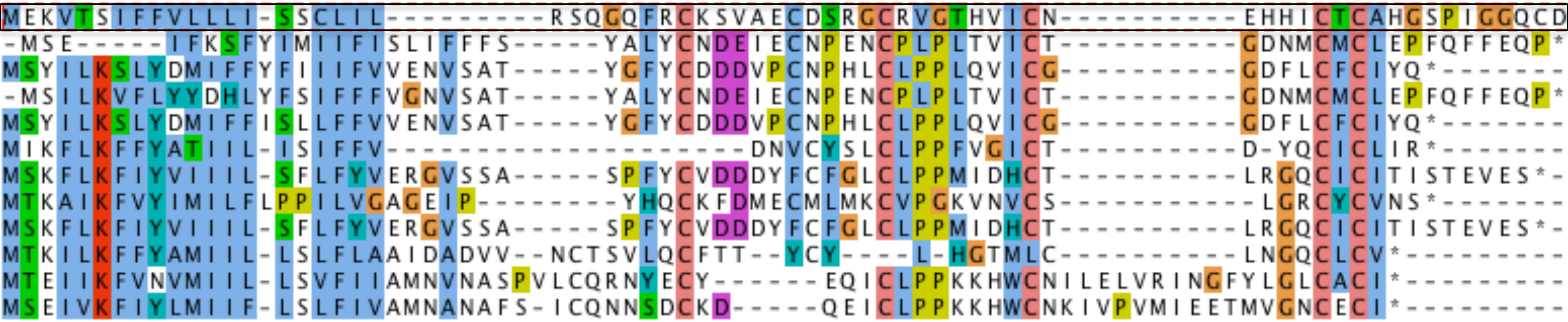

CRP1510

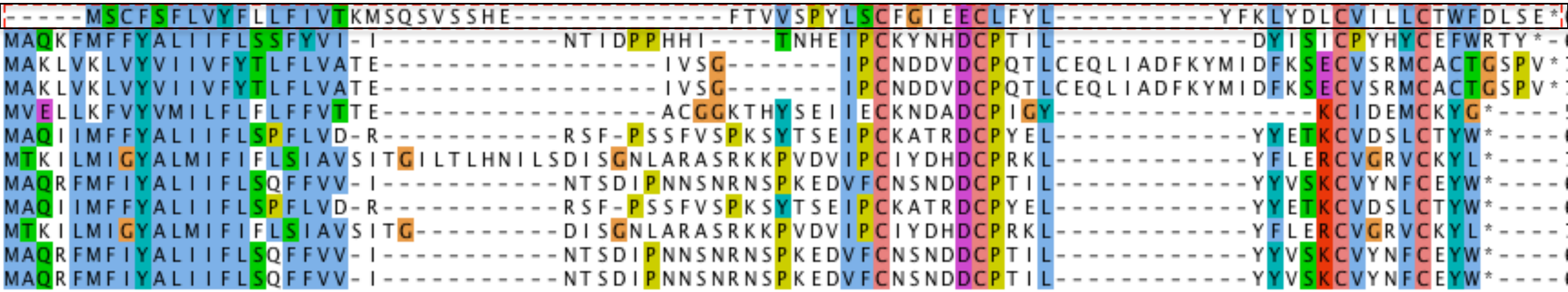

Supplement: Additional file 21 — Figure S7. Sub-class alignments of three Arabidopsis NCRs with Medicago NCRs. In each alignment the first sequence comes from Arabidopsis and the rest all come from Medicago. [file 1471-2105-14-335-S21.pdf]
